# Correlation between intraocular pressure reduction and anterior chamber aqueous flare after micropulse transscleral cyclophotocoagulation

**DOI:** 10.1186/s12886-021-02012-3

**Published:** 2021-06-29

**Authors:** Akitoshi Kimura, Kei-Ichi Nakashima, Toshihiro Inoue

**Affiliations:** Department of Ophthalmology, Faculty of Life Sciences, Kumamoto University, 1-1-1, Honjo, Chuo-ku, Kumamoto, Japan

**Keywords:** Micropulse transscleral cyclophotocoagulation, Glaucoma, Intraocular pressure, Aqueous flare

## Abstract

**Background:**

Micropulse transscleral cyclophotocoagulation (MP-CPC) is a technique that has been approved in recent years to treat glaucoma. MP-CPC causes anterior chamber inflammation; a relationship with reduced intraocular pressure (IOP) has not been reported. Therefore, we analyzed the correlation between IOP and anterior chamber aqueous flare after MP-CPC.

**Methods:**

This retrospective study included 37 eyes of 37 patients who underwent MP-CPC between November 2018 and October 2020. IOP and flare values were measured at 1, 4, and 12 weeks after MP-CPC. Correlations were assessed between the percentage IOP reduction and flare elevation by calculating Spearman’s rank correlation coefficient.

**Results:**

The percentage IOP reduction at 1 week after surgery was correlated with the flare elevation at 1 week after surgery (*ρ* = 0.47, *P* = 0.006). The percentage IOP reduction at 12 weeks after surgery was correlated with the flare elevation at 4 weeks after surgery (*ρ* = 0.53, *P* = 0.006).

**Conclusions:**

A short-term correlation was implied between reduced IOP and flare elevation after MP-CPC.

## Background

Glaucoma is the leading cause of blindness worldwide. The damage to the optic nerve and visual field caused by glaucoma is progressive and irreversible [1]. The only reliable evidence-based treatment for glaucoma is the lowering of intraocular pressure (IOP). Treatment options include medical, laser, and surgical therapies [2, 3]. Traditionally used continuous-wave transscleral cyclophotocoagulation (CW-CPC) is an effective method to lower IOP, but patients can develop serious complications, such as phthisis bulbi [4, 5]. Micropulse transscleral cyclophotocoagulation (MP-CPC) was approved by the US Food and Drug Administration in 2015 as a glaucoma treatment technique. MP-CPC is presumed to be a safe procedure as a result of minimizing thermal coagulation of adjacent tissues and repeating the on/off laser irradiation cycle in microseconds. However, the mechanism of lowering IOP has not been fully explained [4, 5]. MP-CPC causes anterior chamber inflammation; however, a relationship with reduced IOP has not been reported [4–6].

The numbers of cells and the intensity of the flare, reflecting protein concentrations, increase when inflammation occurs in the anterior chamber [7, 8]. A laser flare meter has been developed in Japan to quantitate aqueous flare and cells in the anterior chamber, and the measured values are highly correlated with the numbers of anterior chamber cells and the aqueous flare observed by slit-lamp [7, 8]. Aqueous flare indicates an increase in aqueous humor protein concentrations. When the blood-ocular barriers are disrupted due to inflammation, serum protein leaks into the aqueous humor, and aqueous flare increases [8]. A laser flare meter projects a laser beam into the anterior chamber, and aqueous flare is measured using a photomultiplier to detect scattering of the laser beam by protein in the aqueous humor [7, 8]. These findings motivated us to analyze the correlation between reduced IOP and elevated aqueous flare measured by a laser flare meter after MP-CPC. To the best of our knowledge, this is the first report investigating the relationship between IOP and aqueous flare after MP-CPC. If there were a correlation between inflammation and IOP reduction, it would help elucidate the mechanism of lowering IOP by MP-CPC for the prediction of postoperative IOP.

## Methods

All investigations adhered to the tenets of the Declaration of Helsinki. This study was approved by the Ethics Committee of Kumamoto University. Research information was disclosed to the patients on our website, and the freedom to refuse research use was guaranteed. This retrospective study included consecutive patients with refractory glaucoma who underwent MP-CPC between November 2018 and October 2020 at Kumamoto University Hospital. We excluded the cases in which aqueous flare could not be measured due to poor corneal transparency or poor fixation. We also excluded the case in which additional surgery was performed within 4 weeks. The aqueous flare values of all eyes were measured before and after surgery. MP-CPC was performed using a CYCLO G6™ micropulse P3 probe (IRIDEX, Mountain View, CA, USA) according to the manufacturer’s instructions. Briefly, the machine was preset to a power of 2,000 mW and a duty cycle of 31.3 %. After sub-Tenon’s anesthesia, the MP-CPC probe was applied by continuous sliding for 10–20 s per one-way, 80 s in total per hemisphere, avoiding the filtering bleb and the glaucoma drainage device (all cases were Baerveldt glaucoma implant). After the surgery, 1.5 % levofloxacin eye drops and 0.1 % fluorometholone eye drops were used for 1 week. However, in patients with excess postoperative inflammation, the fluorometholone eye drops were continued for more than 1 week or switched to betamethasone until the inflammation subsided. Patients who underwent additional surgery for reasons such as poor IOP reduction were excluded from the analysis after that time point. The IOP and aqueous flare values were measured preoperatively and at 1, 4, and 12 weeks after surgery. Most IOP readings were measured via Goldman applanation tonometry (GAT). The IOP in two eyes were measured using iCare rebound tonometry (Icare Finland Oy, Helsinki, Finland), because the IOP in these eyes could not be measured by GAT. The aqueous flare values were measured using an FM-700 laser flare meter (KOWA Co., Nagoya, Japan). Visual acuity was measured in the form of decimal visual acuity with the Landolt chart and converted into the logarithm of the minimum angle of resolution (LogMAR) scale, where counting fingers (CF), hand motion (HM), light perception (LP), and no light perception (NLP) were assigned values of 2.1, 2.4, 2.7, and 3.0, respectively [9]. The visual field of patients with good fixation were examined using a Humphrey Field Analyzer (Carl Zeiss Meditec, Dublin, CA, USA). The anterior segment was evaluated using slit-lamp biomicroscopy, and the posterior segment was evaluated using indirect ophthalmoscopy and spectral-domain optical coherence tomography (SD-OCT). History of smoking was queried on the questionnaire. Surgical success was defined as an attained IOP reading between 5 and 21 mmHg and > 20 % IOP reduction at 12 weeks after MP-CPC, compared with the baseline without the addition of other IOP reduction therapies. Correlations between the percentage IOP reduction [(preoperative IOP − postoperative IOP) / preoperative IOP] and elevated aqueous flare (postoperative flare − preoperative flare) were estimated by calculating Spearman’s rank correlation coefficient. A P-value < 0.05 was considered significant.

## Results

Thirty-seven eyes of 37 patients were enrolled in this study. The mean age ± standard deviation (SD) was 70.6 ± 14.4 years, and 20 patients (54.1 %) were male. Sixteen eyes (43.2 %) had exfoliation glaucoma (EXG), nine eyes (24.3 %) had primary open-angle glaucoma (POAG), eight eyes (21.6 %) had uveitic glaucoma (UG), and four eyes (10.8 %) had neovascular glaucoma (NVG) (Table 1). In all, 28 eyes (75.7 %) had a history of cataract surgery, 13 (35.1 %) received trabeculectomy, (5 [13.5 %] of which also underwent Baerveldt tube shunt surgery), 7 (18.9 %) received vitrectomy, 12 (32.4 %) received trabeculotomy, 1 (2.7 %) had CW-CPC, and 8 (21.6 %) had MP-CPC.

**Table 1:** Patient characteristics at baseline

Number of eyes	37
Sex [n (%)]	
Male	20 (54.1)
Female	17 (45.9)
Age (mean ± SD) (years)	70.6 ± 14.4
Glaucoma type, n (%)	
Exfoliation glaucoma	16 (43.2)
Primary open-angle glaucoma	9 (24.3)
Uveitic glaucoma	8 (21.6)
Neovascular glaucoma	4 (10.8)
Preoperative IOP (mean ± SD) (mmHg)	31.8 ± 10.1
Exfoliation glaucoma	31.9 ± 9.0
Primary open-angle glaucoma	25.1 ± 7.0
Uveitic glaucoma	37.5 ± 11.3
Neovascular glaucoma	34.6 ± 12.2
Preoperative aqueous flare value (mean ± SD) (photon counts/ms)	45.0 ± 56.1
Exfoliation glaucoma	26.0 ± 16.1
Primary open-angle glaucoma	38.7 ± 54.8
Uveitic glaucoma	81.6 ± 95.6
Neovascular glaucoma	61.8 ± 33.8
Preoperative LogMAR visual acuity	1.49 ± 0.9
Preoperative HFA mean deviation (mean ± SD) (dB) *n = 4	-27.03 ± 5.4
Smoking, n (%)	
Present	3(8.1)
Past	9(24.3)
Never	25(67.6)

*SD* standard deviation; *IOP* intraocular pressure; *LogMAR* logarithm of minimum angle of resolution; *HFA* Humphrey field analyzer

**Fig. 1:**
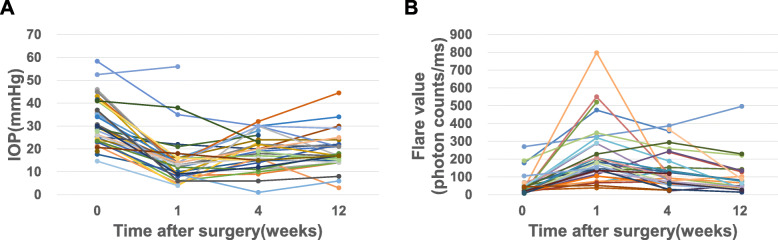
(**A**) Time course of intraocular pressure (IOP) and (**B**) time course of aqueous flare values in all cases

The time course of IOP and the aqueous flare value in all cases are shown in Fig. 1. The mean IOPs ± SD were 14.5 ± 10.5, 19.0 ± 7.5, and 19.0 ± 8.4 mmHg at 1, 4, and 12 weeks after surgery, respectively. The corresponding aqueous flare values were 218.4 ± 165.4, 134.9 ± 103.6, and 97.0 ± 102.2 photon counts/ms, respectively. The mean success rate was 43.2 % at 12 weeks after surgery. By glaucoma type, the mean success rates of EXG, POAG, UG, and NVG group were 43.8 %, 33.3 %, 62.5 %, and 25.0 %, respectively. Complications were observed in 11patients: 5 (13.5 %) had prolonged anterior chamber inflammation that persisted for more than 4 weeks, 1 of whom also had macular edema (2.7 %); 5 (13.5 %) developed hypotony (IOP < 4 mmHg); and 1 (2.7 %) had hypotony maculopathy. Additional treatments were performed in 11 patients, 7 underwent MP-CPC, 1 received Baerveldt tube shunt surgery, 1 had selective laser trabeculoplasty (SLT), 1 had sub-Tenon injection of triamcinolone acetonide for macular edema, and 1 underwent cataract surgery. Details for each glaucoma type are shown in Table 2.

**Table 2:** Success rate, postoperative complications, and additional treatments by glaucoma type

	EXG (*n* = 16)	POAG (*n* = 9)	UG (*n* = 8)	NVG (*n* = 4)	Total (*n* = 37)
Success rate (%)	43.8	33.3	62.5	25.0	43.2
Complications					
Prolonged inflammation	3(18.8 %)	0(0 %)	2(25.0 %)	0(0 %)	5(13.5 %)
Macular edema	1(6.3 %)	0(0 %)	0(0 %)	0(0 %)	1(2.7 %)
Hypotony	4(25.0 %)	0(0 %)	1(12.5 %)	0(0 %)	5(13.5 %)
Hypotony maculopathy	0(0 %)	0(0 %)	1(12.5 %)	0(0 %)	1(2.7 %)
Additional treatments					
MP-CPC	2(12.5 %)	1(11.1 %)	3(37.5 %)	1(25.0 %)	7(18.9 %)
Baerveldt surgery	0(0 %)	1(11.1 %)	0(0 %)	0(0 %)	1(2.7 %)
SLT	1(6.3 %)	0(0 %)	0(0 %)	0(0 %)	1(2.7 %)
sub-Tenon injection of TA	1(6.3 %)	0(0 %)	0(0 %)	0(0 %)	1(2.7 %)
Cataract surgery	0(0 %)	1(11.1 %)	0(0 %)	0(0 %)	1(2.7 %)

*EXG* exfoliation glaucoma; *POAG* primary open-angle glaucoma; *UG* uveitic glaucoma; *NVG* neovascular glaucoma; *MP-CPC* micropulse transscleral cyclophotocoagulation; *SLT* selective laser trabeculoplasty; *TA* triamcinolone acetonide

**Fig. 2:**
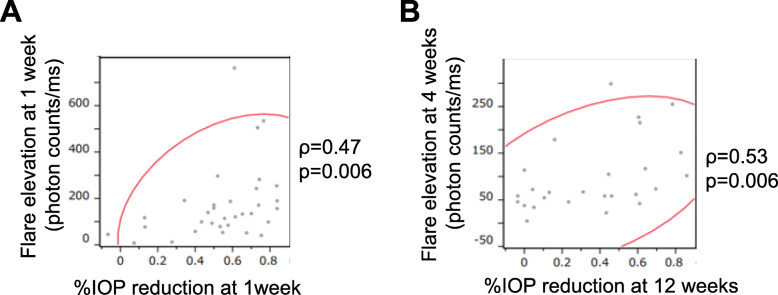
Correlations between the percentage IOP reduction and elevation in the aqueous flare value estimated by Spearman’s rank correlation coefficient. (**A**) Flare elevation at 1 week after surgery was positively correlated with the percentage IOP reduction at 1 week after surgery (*ρ* = 0.47, *P* = 0.006). (**B**) Flare elevation at 4 weeks after surgery was positively correlated with the percentage IOP reduction at 12 weeks after surgery (*ρ* = 0.53, *P* = 0.006)

Flare elevation at 1 week after surgery was positively correlated with the percentage IOP reduction at 1 week after surgery (*ρ* = 0.47, *P* = 0.006; Fig. 2 A). In addition, flare elevation at 4 weeks after surgery was positively correlated with the percentage IOP reduction at 12 weeks after surgery (*ρ* = 0.53, *P* = 0.006; Fig. 2B). We also analyzed each type of glaucoma. Percentage IOP reduction tended to correlate with flare elevation in UG and NVG; however, the sample size was too small to reach any conclusions (Table 3).

**Table 3:** Correlations between the percentage IOP reduction and flare elevation by glaucoma type

	%IOP reduction at 1 week versus flare elevation at 1 week			%IOP reduction at 12 weeks versus flare elevation at 4 weeks		
	ρ	p	n	ρ	p	n
EXG	0.20	0.503	14	0.30	0.405	10
POAG	0.71	0.047	8	-0.43	0.397	6
UG	0.83	0.021	7	0.82	0.023	7
NVG	1	< 0.001	4	1	< 0.001	3

*IOP* intraocular pressure; *EXG* exfoliation glaucoma; *POAG* primary open-angle glaucoma; *UG* uveitic glaucoma; *NVG* neovascular glaucoma

## Discussion

The mean surgical success rate was 43.2 % at 12 weeks after surgery, lower than in previous studies [6, 10]. This may be due to the large number of refractory glaucoma patients with a history of glaucoma surgery in this study and different MP-CPC irradiation methods. A correlation was detected between flare elevation and the percentage IOP reduction at 1 week after surgery. However, no correlation was observed between flare elevation and the IOP reduction at 4 or 12 weeks. These results indicate that the degree of acute intraocular inflammation reflected the IOP reduction at the early stage after MP-CPC, but not at the later stage. MP-CPC may cause intraocular inflammation by coagulating the ciliary body at the early stage, thereby decreasing production of the aqueous humor at that time [11]. However, some studies have reported that MP-CPC does not cause significant damage to the ciliary body because the 810-nm irradiation is emitted in on/off cycling mode to avoid ciliary body destruction. Therefore, there may be a mechanism of IOP reduction other than decreased aqueous humor production [12, 13]. An alternative explanation for the correlation would be increased aqueous outflow by proinflammatory cytokines induced by acute intraocular inflammation. For example, interleukin-1α, interleukin-8, and monocyte chemoattractant protein-1 (MCP-1) increase aqueous humor outflow [14–16]. In general, intraocular inflammation occurs time-dependently, while the reduction in IOP was maintained after MP-CPC. Thus, it would be reasonable to say that the degree of intraocular inflammation at that time did not reflect the reduction in IOP at the later stage.

Flare elevation at 4 weeks after surgery was correlated with the percentage IOP reduction at 12 weeks after surgery, indicating that the degree of inflammation at 4 weeks after surgery may predict IOP control in the future, to some extent. Additionally, the mechanism of IOP reduction by MP-CPC may differ between the acute and chronic stages. However, the actual mechanism remains unclear, so further studies are required. Analysis by glaucoma type showed that a correlation between IOP reduction and flare elevation may be more likely to be observed in UG and NVG. Given that UG and NVG usually have a strong inflammatory response to invasive treatment, it is speculated that a short-term increase in flare value reflects the degree of ciliary body destruction. In addition, UG and NVG tend to have higher levels of proinflammatory cytokines such as MCP-1, which increase aqueous humor outflow, and it is speculated that this may be related to the IOP reduction in the later stage [17, 18]. Further studies with a larger sample size are required.

The limitations of the present study are its retrospective design, small sample size, and short follow-up time after MP-CPC.

## Conclusions

We observed a positive correlation between postoperative reduction in IOP and elevated aqueous flare after MP-CPC. This correlation is more likely to be seen in UG and NVG, which have higher levels of proinflammatory cytokines. Thus, our results suggest that anterior chamber inflammation is associated with IOP reduction by MP-CPC. These findings provide insight into the IOP-lowering mechanism of MP-CPC; however, further studies are required.

## Data Availability

The datasets used and/or analyzed during the current study are available from the corresponding author on reasonable request.

## Abbreviations

IOP: Intraocular pressure
CW-CPC: Continuous-wave transscleral cyclophotocoagulation
MP-CPC: Micropulse transscleral cyclophotocoagulation
GAT: Goldman applanation tonometry
LogMAR: Logarithm of the minimum angle of resolution
CF: Counting fingers
HM: Hand motion
LP: Light perception
NLP: No light perception
SD-OCT: Spectral-domain optical coherence tomography
SD: Standard deviation
EXG: Exfoliation glaucoma
POAG: Primary open-angle glaucoma
UG: Uveitic glaucoma
NVG: Neovascular glaucoma
SLT: Selective laser trabeculoplasty
MCP-1: Monocyte chemoattractant protein-1

## References

[1] Quigley HA, Broman AT (2006). The number of people with glaucoma worldwide in 2010 and 2020. Br J Ophthalmol.

[2] The AGIS Investigators. The Advanced Glaucoma Intervention Study (AGIS): 7. The relationship between control of intraocular pressure and visual field deterioration. Am J Ophthalmol. 2000;130:429–440. 10.1016/s0002-9394(00)00538-9 11024415

[3] Ronan C, Hady S, Iqbal IK (2017). Glaucoma treatment trends: a review. Can J Ophthalmol.

[4] Aquino MC, Barton K, Tan AM (2015). Micropulse versus continuous wave transscleral diode cyclophotocoagulation in refractory glaucoma: A randomized exploratory study. Clin Exp Ophthalmol.

[5] Ndulue JK, Rahmatnejad K, Sanvicente C (2018). Evolution of cyclophotocoagulation. J Ophthalmic Vis Res.

[6] Williams AL, Moster MR, Rahmatnejad K (2018). Clinical efficacy and safety profile of micropulse transscleral cyclophotocoagulation in refractory glaucoma. J Glaucoma.

[7] Ladas JG, Wheeler NC, Morhun PJ (2005). Laser flare-cell photometry: methodology and clinical applications. Surv Ophthalmol.

[8] Tugal-Tutkun I, HerbortCP (2010). Laser flare photometry: a noninvasive, objective, and quantitative method to measure intraocular inflammation. Int Ophthalmol.

[9] Hu X, Pan Q, Zheng J (2019). Reoperation following vitrectomy for diabetic vitreous hemorrhage with versus without preoperative intravitreal bevacizumab. BMC Ophthalmol.

[10] Al Habash A, AlAhmadi AS (2019). Outcome Of MicroPulse Transscleral Photocoagulation In Different Types Of Glaucoma. Clin Ophthalmol.

[11] Carsten H, Beatrix ZI, Jorg K (2011). Long-term reduction of laser flare values after trabeculectomy but not after cyclodestructive procedures in uveitis patients. Int Ophthalmol.

[12] Moussa K, Feinstein M, Pekmezci M (2020). Histologic Changes Following Continuous Wave and Micropulse Transscleral Cyclophotocoagulation: A Randomized Comparative Study. Transl Vis Sci Technol.

[13] Maslin JS, Chen PP, Sinard J (2020). Histopathologic changes in cadaver eyes after MicroPulse and continuous wave transscleral cyclophotocoagulation. Can J Ophthalmol.

[14] Tsuboi N, Inoue T, Kawai M (2012). The Effect of Monocyte Chemoattractant Protein-1/CC Chemokine Ligand 2 on Aqueous Humor Outflow Facility. Invest Ophthalmol Vis Sci.

[15] Birke MT, Birke K, Lutjen-Drecoll E, Schlotzer-Schrehardt U, Hammer CM (2011). Cytokine-dependent ELAM-1 induction and concomitant intraocular pressure regulation in porcine anterior eye perfusion culture. Invest Ophthalmol Vis Sci..

[16] Alvarado JA, Yeh RF, Franse-Carman L, Marcellino G, Brownstein MJ (2005). Interactions between endothelia of the trabecular meshwork and of Schlemm’s canal: a new insight into the regulation of aqueous outflow in the eye. Trans Am Ophthalmol Soc.

[17] Ohira S, Inoue T, Shobayashi K, Iwao K, Fukushima M, Tanihara H (2015). Simultaneous increase in multiple proinflammatory cytokines in the aqueous humor in neovascular glaucoma with and without intravitreal bevacizumab injection. Invest Ophthalmol Vis Sci.

[18] Ohira S, Inoue T, Iwao K, Takahashi E, Tanihara H (2016). Factors influencing aqueous proinflammatory cytokines and growth factors in uveitic glaucoma. PLoS One.

